# Pore-scale modelling and sensitivity analyses of hydrogen-brine multiphase flow in geological porous media

**DOI:** 10.1038/s41598-021-87490-7

**Published:** 2021-04-16

**Authors:** Leila Hashemi, Martin Blunt, Hadi Hajibeygi

**Affiliations:** 1 Faculty of Civil Engineering and Geosciences, Delft University of Technology, P.O. Box 5048, 2600 GA Delft, The Netherlands; 2 Department of Earth Science and Engineering, Imperial College London, London, SW7 2AZ UK

**Keywords:** Hydrology, Mathematics and computing

## Abstract

Underground hydrogen storage (UHS) in initially brine-saturated deep porous rocks is a promising large-scale energy storage technology, due to hydrogen’s high specific energy capacity and the high volumetric capacity of aquifers. Appropriate selection of a feasible and safe storage site vitally depends on understanding hydrogen transport characteristics in the subsurface. Unfortunately there exist no robust experimental analyses in the literature to properly characterise this complex process. As such, in this work, we present a systematic pore-scale modelling study to quantify the crucial reservoir-scale functions of relative permeability and capillary pressure and their dependencies on fluid and reservoir rock conditions. To conduct a conclusive study, in the absence of sufficient experimental data, a rigorous sensitivity analysis has been performed to quantify the impacts of uncertain fluid and rock properties on these upscaled functions. The parameters are varied around a base-case, which is obtained through matching to the existing experimental study. Moreover, cyclic hysteretic multiphase flow is also studied, which is a relevant aspect for cyclic hydrogen-brine energy storage projects. The present study applies pore-scale analysis to predict the flow of hydrogen in storage formations, and to quantify the sensitivity to the micro-scale characteristics of contact angle (i.e., wettability) and porous rock structure.

## Introduction

Renewable energy sources such as wind and solar are intermittent in nature. Therefore, to develop a low-carbon-based energy mix in the future, large-scale storage technologies need to be developed. Hydrogen is an attractive energy storage option, since it has a high specific energy capacity of 120 MJ/kg, and its combustion products are clean. However, hydrogen has a low density of 0.09 kg/m^3^ at standard conditions. As such, large-scale volumes, much beyond the scope of surface-based facilities, are required to store energy in the scale of GWhr to TWhr^1^. Geological formations, such as aquifers, depleted hydrocarbon reservoirs and salt caverns provide ample volumes for storing hydrogen at high pressure (thus high energy densities). These formations conveniently allow for large-scale (G-TWhr) storage of hydrogen gas^2–13^.

Among the potential sites for underground hydrogen storage (UHS), deep saline aquifers, which have been widely considered for $\hbox {CO}_2$ storage^14^, provide significant gas storage capacities. Introducing hydrogen into the subsurface, however, can potentially drive many interactions with the existing fluid in the reservoir and the host rock. Note that experimental studies on sandstone rocks have reported low reactivities with the stored hydrogen in the absence of clay^2,4^.

The feasibility of UHS has become an attractive subject only in recent years, while the initial studies go back to 1970s. In 1979, Foh et al. published their final techno-economic report for UHS in the US^15,16^. Later, as shown in Table 1, several UHS field and research projects have been established around the world. More information regarding these projects is provided in the supplementary materials.

**Table 1:** Worldwide underground hydrogen storage projects^2,15^.

Type	Country	Location
Salt Cavern	The UK	Tesside
	The US	Moss Bluff
		Spindletop
		Clemens
	Germany	Bad Lauchstdt
		Kiel
Aquifer	Germany	Ketzin
	France	Beynes
	Czech	Lobodice
	Russia	Kasomovskoie
Depleted gas reservoir	Argentina	Hychio

More information is provided in the supplementary materials.

The economic, societal, and legal aspects of UHS have been addressed in several projects, including H2STORE, HyUnder, ANGUS+, UndergroundSunStorage, Road2HyCOM, and projects in the US^2,15,17^. These studies classify potential sources of hydrogen loss and reactivities in three major categories: (1) Leakage through cap rock and borehole, (2) bio- and geo-chemical reactions, and (3) diffusivity of hydrogen into the brine. All these important aspects are briefly revisited below.

Characterisation of the sealing properties of the cap rock and borehole zone is essential for site selection and operational conditions^17,18^. For instance, in salt caverns, as a rule of thumb, the range of gas operation pressure should be between 24% to 80% of the overburden pressure^19^. The upper bound is set to avoid creating fractures, while the lower one is determined based on maintaining the injectivity of the reservoir^19^. In porous reservoirs, appropriate production rate is a vital parameter to avoid coning of aquifer brine into the well perforation zone^3,4^. Furthermore, as shown in HyINTEGER, another source of hydrogen leakage can be through the well borehole and casing materials^13^.

Bio-chemical and geo-chemical reactions also stand as key factors in the UHS studies. More specifically, stored hydrogen in the reservoir can be consumed by chemical reactions with the host rock and pre-existing fluids. Further consequences of these reactions are changes in porosity and permeability by dissolution or precipitation of minerals or growing biomass^2,15,20^. Therefore, in many UHS projects (i.e., H2STORE, ANGUS+, UndergroundSunStorage, projects in France) these reactions and their consequences were investigated in detail^9,15^. Most of these studies report very limited geochemical interactions, especially at moderate conditions (reservoir pressure and temperature)^2,5,6,21–24^. However, for pyrite-bearing rocks, hydrogen-reductive activities might be considerable, even at low temperatures, which can produce highly-toxic H_2_S gas^20,24^. Furthermore, bio-reactions are known to be the main factor contributing to hydrogen loss^2,9,12,20–23,25,26^. Four common microbial reactions in the context of underground hydrogen storage are methanogenesis, acetogensis, sulfate-reduction, and ferric-reduction that produce methane (CH_4_), acetic acid (CH_3_COOH), hydrogen sulfide (H_2_S), and iron oxide (Fe_3_O_4_), respectively^2^.

Finally, like many other gases, solubility and diffusion and fingering of injected hydrogen into the reservoir brine can be another source of hydrogen loss in geological formations. Even though cushion gas (e.g., $\hbox {N}_2$) is expected to prevent (at least most of) the stored hydrogen from reaching the brine, it is still important to quantify the transport properties of H_2_-brine, so to appropriately design the system when $\hbox {H}_2$ and brine come to contact in the reservoir. The dissolved hydrogen in the brine also changes the thermodynamic equilibrium conditions, which can change the pH and increase the chemical reactivity^2,20,27^. Experimental and numerical studies have been conducted to quantify the dissolution of hydrogen into brine (listed in the supplementary materials). The dissolution is found to depend on pressure, temperature, and salinity^6^. Because the dissolution and diffusion coefficients (listed in the supplementary materials) are small, the solubility of hydrogen into the brine is reported to be minimal, i.e., about 1–3%/year in aquifers^15,18,28,29^ and 0.1-3% in depleted gas reservoirs^20,22^.

In several studies such as H2STORE, ANGUS+, UndergroundSunSrotage, HyUnder, American and French projects, reservoir-scale simulations were performed to estimate the storage efficiency. Except one experimental study at a specific condition for the storage projects in France^2^, there exist no other experimental studies to characterise H_2_-brine transport properties (i.e. capillary pressure and relative permeability). One group of studies have used literature data from natural gas (methane) reservoirs^3,20^. Alternatively, another group of studies have used empirical functions such as van Genuchten and Brooks & Corey for the hydrogen-brine system^4,30^. It is therefore essential to critically analyse the pore-scale transport dynamics of the hydrogen-brine system, and report the range of relative permeability and capillary pressure suitable to simulate this complex process at the scale of the storage site, specially in the absence of robust experimental data. It is also important to investigate whether the use of classical hydrocarbon-based functions is justified, in the new context of hydrogen-brine systems.

This paper reports the first pore-scale-based effective functions to consistently describe hydrogen-brine transport at the reservoir scale. It also reports the sensitivity of the system with respect to the uncertain parameters, and indicates the key parameters to characterise the system accurately for a given storage scenario. This study also accounts for cyclic hysteretic physics, to appropriately analyse primary drainage (hydrogen injection), secondary imbibition (hydrogen withdrawal) and secondary drainage (hydrogen re-injection), which are all important and in some aspects unique (e.g. cyclic transport), for seasonal $\hbox {H}_2$ storage in aquifers.

The rest of the paper is organised as follows. Next, the pore-network methodology is briefly revisited, emphasising novel developments, specific to the H_2_-brine system, including hysteretic contact angles and relevant parameters obtained by processing the existing experiment in the literature. Then results are presented for a systematic study, in which cyclic transport is upscaled to find relative permeability and capillary pressure as a function of saturation. This section also elaborates the ranges of H_2_-brine upscaled functions, their hysteretic behaviour through cyclic transport, and whether empirical functions in the literature are valid. Note that the supplementary materials provide complementary data sets that were left out of the main manuscript, and a list of all UHS projects.

## Methods

Multiphase flow properties in subsurface reservoirs can be predicted by several methods including laboratory measurements and numerical simulations^31–36^. Experimental approaches are costly and time consuming, and are found for a limited samples and conditions^31^. Numerical modelling and simulations are therefore crucial to complement the laboratory studies to allow for wider range of studies and sensitivity analyses.

In this study, the most computationally-efficient pore scale approach, which is called quasi-static pore network modelling “PNM”, was used to simulate fluid flow of UHS at the pore-scale and to estimate the macro-scale properties, i.e., capillary pressure and relative permeability. Quasi-static PNM is an appropriate method for the capillary-dominated flow regime where the capillary number is less than $$10^{-4 } $$^37^. This is a common range for immiscible two-phase flow in many subsurface applications. All the simulations in this paper were implemented by using an open-source software from Imperial College London, called “pnflow”^38^, which is validated with experimental results for hydrocarbon reservoirs^37,39^.

Below, the key components of pore-network modelling are first presented. Then the specific developments relevant for H_2_-brine transport are discussed. Detailed information about the well-developed pore-network modelling approach can be found in the literature^31^. Here, for the sake of brevity, we focus on new developments relevant for H_2_-brine systems.

**Figure 1:**
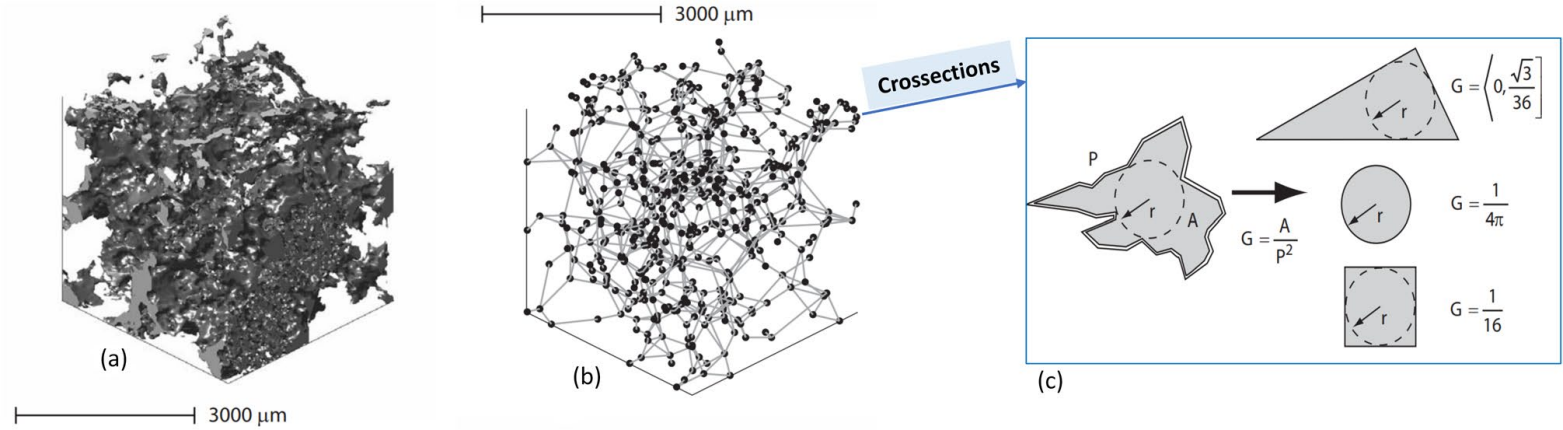
(**a**) 3D image of a sandstone along with (**b**) a topologically equivalent network representation (**c**), and categorizing the cross-section of elements based on the shape factor (G)^31,37^.

**Figure 2:**
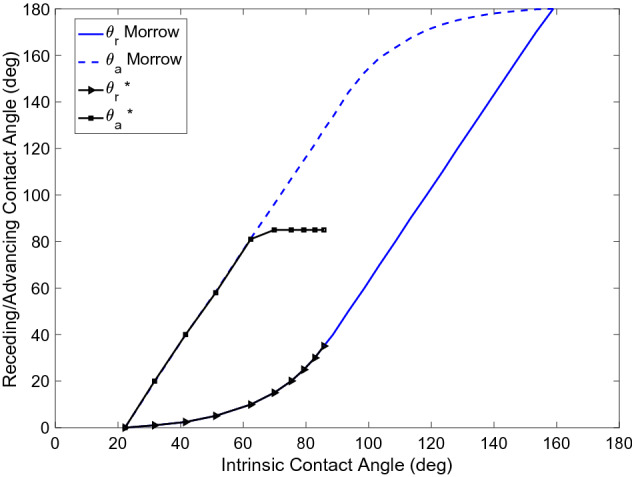
Relationship between receding $\theta _r$ and advancing $\theta _a$ contact angles as functions of intrinsic contact angle $\theta _i$, based on the literature^37,46^. *Indicates the modified relationship, which is defined in this work, to maintain water-wet rock conditions at all times.

**Figure 3:**
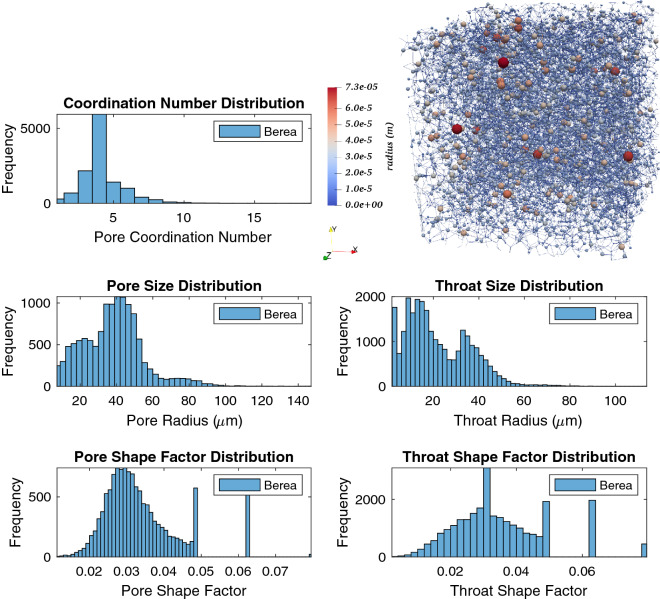
Illustration of the pore network model characteristics for Berea sandstone^47^.

- Pore-Network Modelling (PNM) description
  PNM uses a topologically and geometrically equivalent network of a rock sample to predict capillary pressure and relative permeability by simulating fluid flow through the elements of the network. Processing 3D micro-CT images of a core plug provides information about the network and its elements including radius, volume, length, coordination, clay volume, etc.^40,41^. The elements with larger volume represent “pores” and the elements which connect the pores are called “throats”^31,42^. Figure 1 shows an illustration of a 3D image of a sandstone and its extracted pore network^37^.
  All the elements are uniform ducts with various cross-sectional shapes which are classified into circle, square and triangle shape factors *G*, which is defined as $A/P^2$, where *A* is the cross-sectional area and *P* is the perimeter length^31,43^. Figure 1 presents an illustration of pore shapes. Most of the earliest network models assumed circular cross-sections for simplicity^44^. One major disadvantage of a circular shape is that it cannot accommodate more than one fluid in a stable configuration in a single pore. Therefore, it does not allow for any films or layers of additional phases to be formed during displacement. In contrast, it can be clearly seen from thin section images of rocks that real pore shapes are very irregular and have many corners. Experimental studies have shown that in pores with angular cross sections, the wetting phase can occupy the corners with the non-wetting phase in the centre. These corner wetting layers provide additional phase connectivity and hence have an impact on trapping. Thus, networks that have pore elements with angular cross sections provide the opportunity to predict experiments where corner flow is crucially important.
  In a quasi-static network model the capillary pressure is the driving force that determines the saturation evolution in the model. This quantity is changed gradually and, for each capillary pressure value, the equilibrium position of the fluid-fluid interfaces is determined. The displacements or the changes of fluid configurations occur sequentially, according to the entry capillary pressure criteria and the connectivity of each fluid to the inlet and outlet of the network. Since the network is initially saturated with the wetting phase, displacement process starts with primary drainage followed by secondary imbibition and finally secondary drainage to simulate cyclic injection and withdrawal. The details of the fluid-flow simulation procedure and governing equations have been explained in the literature^37^.
- Fluid and rock parameters
  In the available experimental report for hydrogen-brine system, Yekta et al.^2,45^ used sandstone rocks from the Buntsandstein formation (Vosges level). Two core flooding tests were performed under conditions representative of shallow and deep aquifers. The properties of the rock and the fluids are given in Table 2.
  In this work, we define the base-case for the H_2_-brine system as the one obtained based on the experiment described previously^2,45^. Since the experimental data for hydrogen-brine is limited to only the primary drainage cycle (initial injection of H_2_), to estimate advancing contact angles in all models, the Morrow relationship, as shown in Fig. 2, was used. However, for receding angles more than approximately 12$^\circ$, if one follows the Morrow curves, the corresponding advancing contact angle becomes bigger than 90$^\circ$. This shows the rock becomes hydrogen-wet, which is very unlikely to be realistic. To resolve this challenge, in this study, the maximum advancing contact angle is, therefore, set to 85$^\circ$, as shown in Fig. 2. This modification guarantee that hydrogen is the non-wetting phase in all cases. Additionally, a uniform distribution of contact angle (a constant value in all pores) is employed for all the simulations in this paper. Also, as for the rock, relatively homogeneous Berea sandstone is considered, unless otherwise stated. The extracted network data is illustrated in Fig. 3^47^.

**Table 2:** Reported fluid and rock properties for H_2_-brine experimental primary drainage tests^2,45^.

**Rock**	Formation		Porosity (%)				Permeability (mD)	
	Vosges-sandstone		19.8				46	
**Fluid**	Depth	$\upsigma _{\mathrm{H}_2,\mathrm{brine}}$ (mN/m)	$\upmu$ (Pa.s)$\times$10^6^		$\uprho$ (kg/m^3^)		cos($\uptheta$_r_)	$\uptheta _{r}$ (degrees)
			H_2_	brine	H_2_	brine		
	Shallow (5 MPa, $20\,^\circ$C)	51	8.94	999	5.6	1000.5	0.93	21.56
	Deep (10 MPa, $45\,^\circ$C)	46	9.54	597	7.2	994.5	0.82	34.9

**Figure 4:**
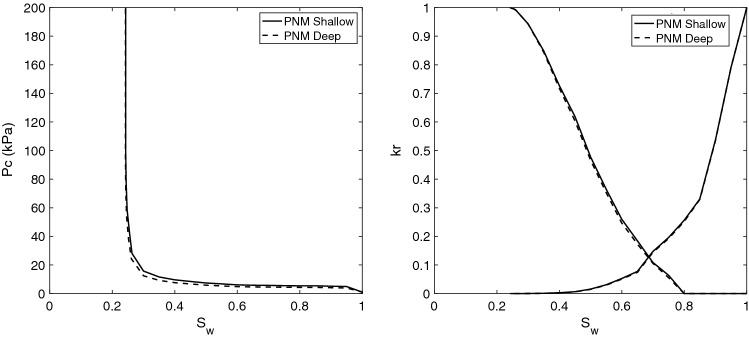
Capillary pressure (left) and relative permeabilities (right) for the base case, i.e., for Berea sandstone using the experimentally-relevant fluid properties of $\theta _a = 85^\circ$, $\theta _r = 21.56^\circ$ and $\sigma = 51$ (mN/m). The base case corresponds to the Shallow formation of Table 2. Also shown are the results corresponding to the fluid properties of the Deep formation.

The base case scenario for our analyses is defined as follows. The base rock is considered to be the Berea sandstone with the characteristics presented above. The receding contact angle and the interfacial tension for H_2_-brine in the base case are defined following the available experimental data at the core scale^2,45^. The receding contact angle, assumed to be constant across the micro-scale sample, was found by fitting to the measured capillary pressure. As presented in the literature, this results in $\theta _r = 21.56^\circ$ and $\sigma = 51$ [mN/m], as shown in Table 2. Based on the modified-Morrow hysteresis curve, Fig. 2, the advancing contact angle for the base case is set as $\theta _a = 85^\circ$. Using these data sets, the relative permeability and capillary pressure for the base case is presented in Fig. 4. Note that the base case properties correspond to the Shallow formation reported in Table 2. Moreover, the results corresponding to the fluid properties of the Deep formation are also presented in Fig. 4. Consistent with the reported experiments, our pore-network model results also confirm insensitivity towards different pressure and temperature values of Shallow and Deep formations. This is because we only have a small change in interfacial tension, which affects the capillary pressure, and no change in advancing contact angle, leading to identical relative permeabilities in the two cases.

What comes next is a systematic analysis and quantification of uncertainty for cyclic transport and within the parameter range of the available experimental data^2,45^. In addition, we investigate whether empirical Corey functions^30^ are acceptable for upscaling hydrogen-brine transport. Rock and fluid parameters are listed in Table 3. Note that to differentiate the effect of different rock parameters such as clay volume and coordination number (i.e., the number of throats connected to a pore), statistical models were generated^47^.

**Table 3:** Parameters used in the simulations.

	Category	Parameter	Range
Fluid properties	Wettability	1. Contact angle	$\theta _r \in [5-30]^\circ$, $\theta _a \in [58-85]^\circ$
Rock properties	Extracted network	2. Structure of sandstones	Table 5
		3. Structure of carbonates	
	Generated network statistics	4. Clay volume	[0–30]%
		5. Averaged coordination number	[3-6]

## Results

Hydrogen-brine transport for energy storage applications is unique in some aspects. Firstly, the upscaled transport functions need to be benchmarked against empirical functions employed in the literature for other gas-brine systems, especially Corey functions. Our first test case addresses this important aspect. Then, the cyclic nature of the storage application, requires the simulation of cycles of injection and production. This leads to adding the secondary drainage into the pore-network modelling framework. The second test cases deals with this important aspect, and quantifies the hysteretic nature of the upscaled functions around the base case scenario. Lastly, uncertainty in the fluid and rock properties needs to be investigated, to find their impact on the upscaled multiphase flow functions. The last two test cases address the uncertainty in the fluid and rock properties, respectively. With these studies we aim to highlight the key aspects of hydrogen-brine transport for energy storage. A summary of the test cases is given in the list below.

- *Test Case 1* Benchmarking pore-network upscaled functions with those obtained with the widely-used Corey equation.
- *Test Case 2* Hysteretic upscaled transport functions for cyclic transport
- *Test Case 3* Impact of fluid properties on the hysteretic upscaled functions
- *Test Case 4* Impact of rock properties on the hysteretic upscaled functions

### Test Case 1: Benchamrking pore-network modelling (PNM) and Corey functions

**Figure 5:**
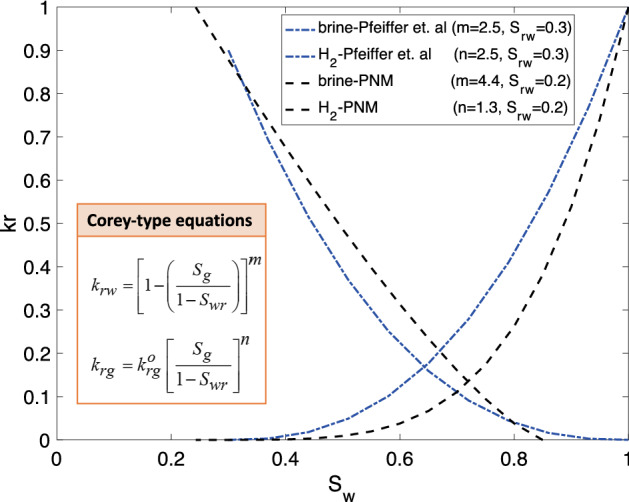
Benchmarking H_2_-brine relative permeability values for primary drainage between PNM and Corey functions with exponents that were used in the literature^30^.

Figure 5 shows the relative permeabilities for hydrogen injection into brine-saturated rock. Since in the literature no hysteresis effects were considered^30^, the PNM results are also plotted only for primary drainage. The best fit to the PNM results, constrained with Corey equation parameters^48^, resulted in different values for the exponents of hydrogen and brine, 1.31 and 4.36 respectively. However, in the literature the constant exponent of 2.5 for both hydrogen and brine has been used^30^. As such, the PNM-based studies indicate that different exponents for hydrogen and brine need to be considered. This makes physical sense, since the exponents are related to pore structure and wettability and are higher for the wetting (brine) phase as it fills the smaller pores, as opposed to lower exponents (higher relative permeabilities) for the non-wetting phase (hydrogen) that preferentially occupies the larger regions of the pore space.

### Test Case 2: Hysteretic upscaled transport functions for cyclic transport

**Figure 6:**
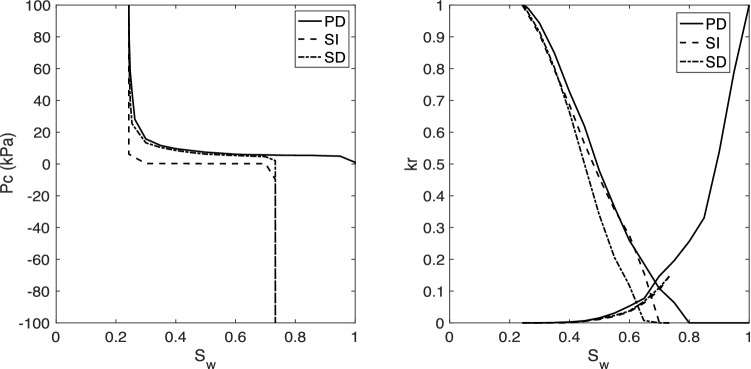
The impact of cyclic transport on capillary pressure and relative permeabilities for primary drainage (PD), secondary imbibition (SI), and secondary drainage (SD).

In real-field hydrogen storage projects, there are repeated cycles of injection and withdrawal. As such, both primary and secondary drainage processes will occur in addition to secondary imbibition. To investigate this, Fig. 6 shows the impact of cyclic injection and withdrawal of hydrogen on capillary pressure and relative permeability. Note that, as discussed in the Methods Section, the modified Morrow values for advancing and receding contact angles have been used. Compared with primary drainage, secondary drainage shows lower relative permeability values at the same saturation. This is due to disconnection of the hydrogen phase after secondary imbibition, as well as the presence of trapped wetting-phase (brine) in some pores. Also, the remaining hydrogen phase, after secondary imbibition, leads to a decrease of capillary pressure values for the secondary drainage stage. Additional cycles of injection and production of hydrogen into the network of Berea sandstone are also investigated and provided in the supplementary material. Our observations indicate that the hysteresis effect stays the same for subsequent drainage and imbibition displacement, after the first injection-production cycle.

### Test Case 3: Impact of fluid properties on the hysteretic upscaled functions

Among the fluid properties, wettability is found to have major impacts on the upscaled functions. The findings are discussed in two separate parts as follows.

**Figure 7:**
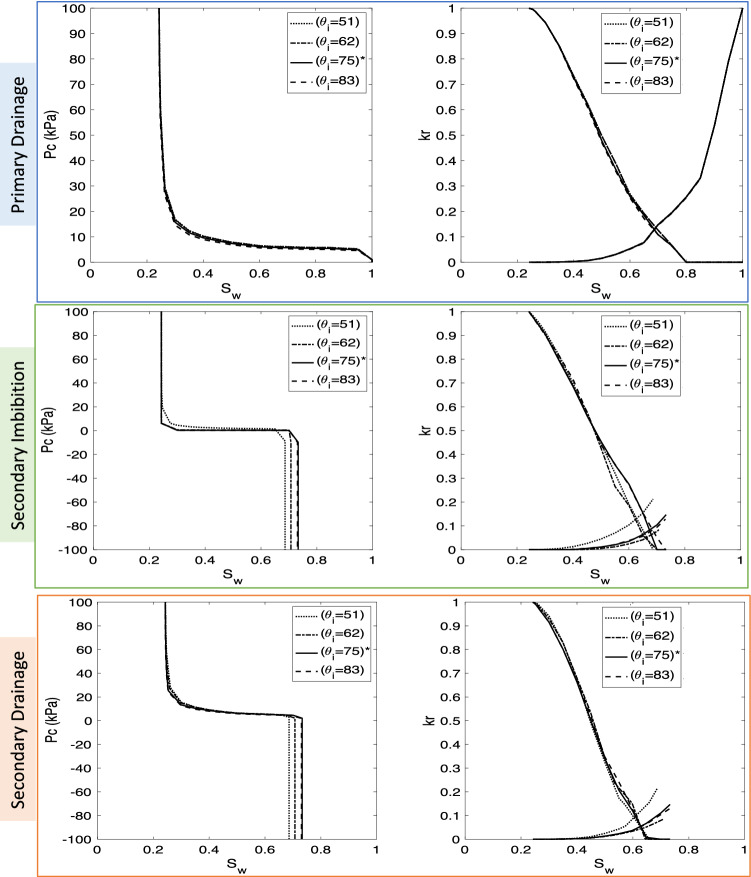
Sensitivity analysis for the H_2_-brine system for primary drainage, secondary imbibition, and secondary drainage due to changing receding and advancing contact angles. $\theta _i$ indicates the intrinsic contact angle, and *indicates the base-case which corresponds to the experimental data reported in the literature^2^, i.e., $\theta _r = 21.56^\circ$ and $\theta _i = 75^\circ$.

**Figure 8:**
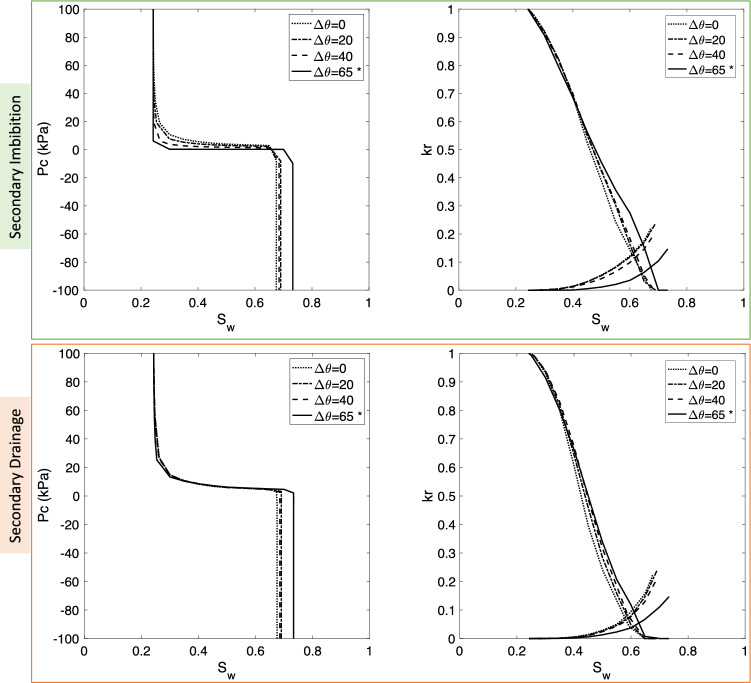
Effect of advancing contact angle on flow properties of H_2_-brine for secondary imbibition. Note that the receding contact angle is fixed at $21.56^\circ$. * indicates the base-case which corresponds to the modified Morrow relationship as shown in Fig. 2.

1. *Effect of wettability*
  The range of simulated contact angles for H_2_-brine is given in Table 4. Simulation results are shown in Fig. 7. Note that increasing contact angles (which are less than 90$^\circ$) changed the wettability of system and made it less water-wet. Since the receding contact angles varied between 5$^\circ$ to 30^o^, there is no significant effect on the results for primary drainage. However, during secondary imbibition and secondary drainage, increasing contact angles resulted in a smaller amount of trapped hydrogen (lower capillary pressure). Also, the maximum relative permeability of the brine phase is shown for the strongly water-wet system with $\theta _i = 51^\circ$. Relative permeabilities of hydrogen during secondary drainage are mostly similar. However, during secondary imbibition, increasing contact angles towards neutral wettability (i.e., when intrinsic contact angles are close to 90$^\circ$) increases the relative permeability of hydrogen. This is due to the reduced amount of trapped hydrogen.
2. *Effect of the difference between advancing and receding contact angles, i.e.*, $\Delta \theta =\theta _a-\theta _r$, *with* $\theta _r = 21.56^\circ$
  In this set of tests, all the parameters of the base-case remained constant except the advancing contact angle. Figure 8 shows the impact of the difference between receding and advancing contact angles on capillary pressure and relative permeabilities. The maximum trapped hydrogen was observed for the case with equal advancing and receding contact angles. For the higher advancing contact angles rock becomes less water-wet. Therefore, relative permeability of hydrogen increases. There is also less trapping of hydrogen.

**Table 4:** Fluid and rock properties used for the wettability sensitivity analysis, by changing advancing ($\theta _a$) and receding ($\theta _r$) contact angles.

Formation		Dimensions (mm$^{3}$)	No. of pores	No. of throats	Porosity (%)	Permeability (mD)
**Rock**						
(big) Berea sandstone		$3\times 3\times 3$	12349	26146	18.33	2551.6

Test no.	$\upsigma _{\mathrm{H}_2,\mathrm{brine}}$ (mN/m)	$\theta _r$ (degrees)	$\theta _a$ (degrees)	$\theta _i$ (degrees)	Viscosity ratio	Density difference (kg/m$^{3}$)
**Fluid**						
1	51	5	58	51	111.745	994.9
2	51	10	81	62	111.745	994.9
3 *	51	21.56	85	75	111.745	994.9
4	51	30	85	83	111.745	994.9

*Indicates the base-case from the literature^2^.

### Test Case 4: Impact of rock properties on the hysteretic upscaled functions

**Figure 9:**
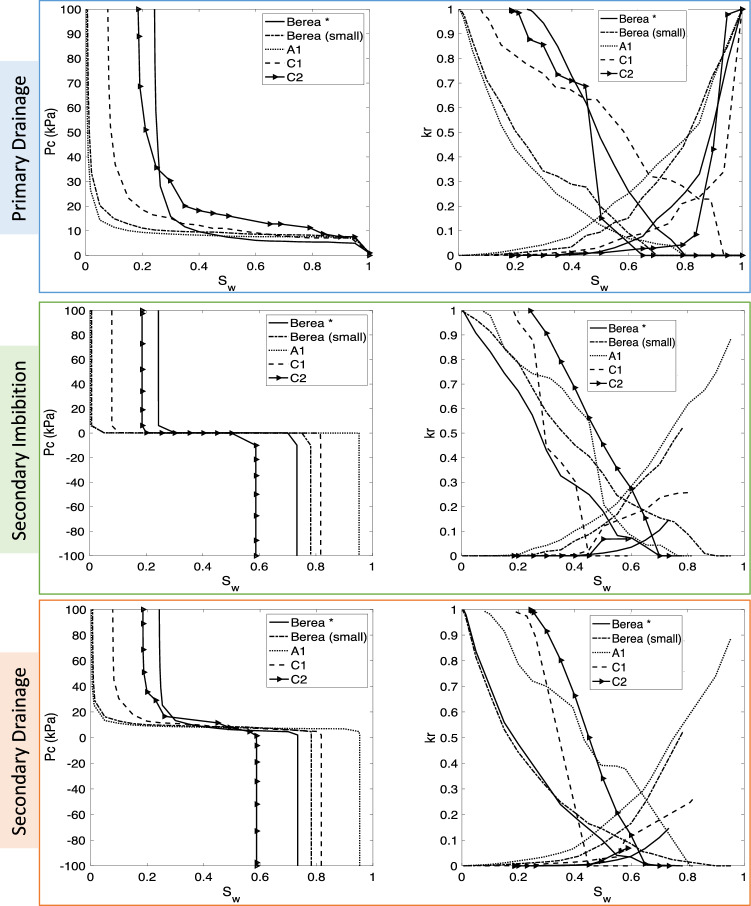
Sensitivity analysis of H_2_-brine system for different rock types. *Indicates the network that was used for the base-case of simulations.

Rock structure has an important role in the transport behaviour of fluids. To study changes in the saturation-dependent functions for various rock types, five extracted pore network models from images of sandstones and carbonates were used^47^. The properties of these samples and the system of the fluids (H_2_-brine) are given in Table 5. Moreover, detailed rock structures for all samples are provided in the supplementary materials. The outcome of the simulations are shown in Fig. 9. Among the three sandstone models, small Berea and A1 showed almost zero water saturation trapped after injecting hydrogen which implies that they contain no clay. Similar patterns are observed for flow properties during primary drainage. Sample A1 which had the highest porosity and absolute permeability had the smallest residual hydrogen saturation and, as expected the highest values for the relative permeability of brine in secondary imbibition. Comparison of the two models from carbonate reservoirs indicates the impact of a complex pore structure. Sample C2 has the smallest pores and restricted connectivity with therefore the highest capillary pressures during all displacement cycles and the highest residual (trapped) hydrogen saturation after secondary imbibition.

**Table 5:** Fluid and rock properties for studying the effect of rock structure on the upscaled transport functions.

	Type	Model name	Dimensions (mm$^{3}$)	No. of pores	No. of throats	Porosity (%)	Permeability (mD)	Ave. coordination number
Rock	Sandstone	Berea*	$3.000\times 3.000\times 3.000$	12349	26146	18.33	2551.6	4.19
		Berea (small)	$2.138\times 2.138\times 2.138$	6298	12545	19.60	1111.0	3.91
		A1	$1.155\times 1.155\times 1.155$	3393	11479	42.85	8125.5	6.65
	Carbonate	C1	$1.140\times 1.140\times 1.140$	4576	6921	24.67	1164.8	2.98
		C2	$2.138\times 2.138\times 2.138$	8508	10336	15.84	161.61	2.37
Fluid	Phases	$\upsigma _{\mathrm{H}_2,\mathrm{brine}}$ (mN/m)	$\theta _r$ (degrees)	$\theta _a$ (degrees)	Viscosity ratio		Density difference (kg/m^3^)	
	H$^{2}$-brine	51	21.56	85	111.745		994.9	

*Indicates the base-case simulations.

**Figure 10:**
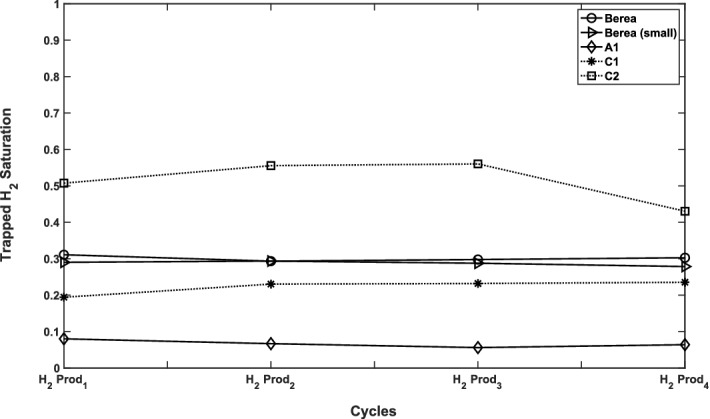
Sensitivity analysis of saturation of trapped hydrogen after 4 cycles of production for different rock types with advancing ($\theta _a = 81^\circ$) and receding ($\theta _r = 10^\circ$) contact angles and fluid properties of the base-case.

We define the maximum trapped hydrogen saturation as the one found after production corresponding to a capillary pressure $P_c = -100$ kPa. Figure 10 shows that many injection-production cycles do not change the maximum amount of produced hydrogen significantly. However, the maximum trapped hydrogen is affected by the rock structure. Characterisation of these models (as provided fully in the supplementary material) indicates that lower connectivities and the smaller pores in model C2 is indeed the reason behind its the highest residual hydrogen saturation, among all samples. On the other hand, model A1 with the maximum average coordination number and high permeability allows for the highest hydrogen production (i.e., minimum residual hydrogen saturation). In addition, the two models of Berea sandstone, with similar characteristics but different sample sizes, have almost equal storage efficiency.

To differentiate the effect of different rock parameters such as clay volume and coordination number, some statistical models were generated using an open source software^47^. The percentage of clay volume in the network directly changes the trapped water saturation after primary drainage, but the patterns of capillary pressures and relative permeability remain similar. Increasing the coordination number reduces capillary entry pressures and trapped hydrogen saturation during secondary imbibition. Moreover, its effect on relative permeabilities becomes significant during displacement of hydrogen by water: higher coordination number facilitates flow and suppresses trapping. Full details are presented in the supplementary material.

## Conclusions

$\hbox {H}_2$-brine transport properties are quantified at the continuum scale through capillary pressure and relative permeability. These functions were predicted based on pore network modelling (PNM) which simulates the pore-scale displacement of fluids. Through several systematic studies we first benchmarked the PNM fluid parameters with existing experimental data. This allowed us define a meaningful base case configuration. The brine remains as the wetting phase. When the relative permeabilities are fit to a power-law type empirical model, the exponents are higher than for the non-wetting, hydrogen, phase. This is due to the fact that brine tends to occupy the smaller regions of the pore space. In addition, cyclic hydrogen storage in subsurface geological formations imposes hysteretic behaviour. This effect, which needs to be considered for accurate transport simulations at reservoir scales, was studied for both capillary pressure and relative permeability. Systematic sensitivity analyses demonstrated that both capillary pressure and relative permeability are sensitive to contact angles as a representation of wettability. The pore structure also is another key determinant of multiphase flow properties. More precisely, using different image-based models of sandstones and carbonates resulted in very different relative permeabilities and capillary pressures for hydrogen-brine multiphase transport. This sensitivity analysis was further enriched by detailed characteristics of the studied rock samples. Also, various clay percentages affected the end-point values for drainage and imbibition cycles. Coordination number which quantifies the network connectivity also had significant effect on the residual saturation of the non-wetting phase (hydrogen) after secondary imbibition.

The present study reports the base line for further research on the characterisation of $\hbox {H}_2$ transport properties while highlighting the need for further investigations in a laboratory environment.

The modelling data and upscaled functions are all made available open access at https://gitlab.tudelft.nl/ADMIRE_Public/PoreScale_H2repository.

## Supplementary Information

Supplementary Information 1.
