# Supplementary material for: Pore-scale modelling and sensitivity analyses of hydrogen-brine multiphase flow in geological porous media
Source: Sci Rep. 2021 Apr 16;11:8348. doi: 10.1038/s41598-021-87490-7 (PMC8052453; doi:10.1038/s41598-021-87490-7)
Supplement: Supplementary file 1 — Supplementary Information 1. [file 41598_2021_87490_MOESM1_ESM.pdf]

Supplementary Information
Pore-scale modelling and sensitivity analyses of hydrogen-brine multiphase flow in geological porous media

Leila Hashemi[1], **Martin J. Blunt**[2], **and Hadi Hajibeygi**[1,*]

[1]Delft University of Technology, Faculty of Civil Engineering and Geosciences, Delft, P.O. Box 5048, 2600 GA, The Netherlands.
[2]Imperial College London, Department of Earth Science and Engineering, London, SW7 2AZ, United Kingdom.
[*]h.hajibeygi@tudelft.nl

ABSTRACT

Table 1. Research projects related to UHS[1,2].

Name of project	Description
H2STORE	Duration: started in 2012[2]<br>Contributed country: Germany[2]<br>Participant(s): the German Federal Ministry of Education and Research (R&D program of Energy Storing), Coordinator: FSU Jena. Partnerships: TU Clausthal, EFZN, GFZ Helmholtz-Centre Potsdam, and LEMTA-University of Lorraine, Nancy[2]<br>Note:This project was related to geohydraulic, mineralogical, geochemical and biogenic reactions, by focusing UHS in depleted gas reservoirs, meanwhile aquifers were the other option for storing[2]
InSpEE	Duration: started in 2012[2]<br>Contributed country: Germany[2]<br>Participant(s):the German Federal Ministry of Education and Research (R&D program of Energy Storing), Coordinator: KBB Underground Technologies. Partnerships: BGR (Federal Institute for Geosciences and Natural Resources); Leibniz University of Hannover[2]<br>Note: "Informational Systems in Salt Structures" was related to intelligent computer-assisted control of storage behavior[2]
HyUnder	Duration: 2012 - 2014[2]<br>Contributed country: EU (Germany, Netherlands, Romania)[2]<br>Participant(s): CENEX (Centre of Excellence of Low Carbon and Fuel Cell Technologies), CEA (Commissariat a l'Energie atomique et aux energies alternatives), DEEP Underground Engineering, ECN (The Energy Research Centre of the Netherlands), E.ON Gas Storage, HINICIO, LBST (Ludwig Bolkow Systemtechnik), KBB Underground Technologies, HYRO (National Hydrogen and Fuel Cell Centre from Romania), and Shell Global Solutions International B.V., Solvay[2].<br>Note: This was the first European R&D project about UHS by focusing on engineering and economic aspects[2].
Hychio	Duration: started in 2006 till present[1]<br>Contributed country: Argantina[1]<br>Participant(s): Argentina[1]<br>Note: This was one of the first field test of UHS in depleted gas reservoirs. So, its pilot started since 2009 which received hydrogen from the electrolysing of water. Then pure hydrogen is combined with natural gas and by combusting that, the energy is produced again. In addition other product of electrolyse, Oxygen is sold to the gas market. This project has been working successfully so far[1].
ANGUS+	Duration: started in the mid of 2013 and finished in 2015[1]<br>Contributed country: Germany[2]<br>Participant(s):the German Federal Ministry of Education and Research (R&D program of Energy Storing), Coordinators: Christian-Albrechts University of Kiel. Partnerships: Deutsches Helmholtz GeoForschungs Zentrum (GFZ), and Helmholtz Centre for Environmental Research-UFZ; Ruhr-University Bochum[2,1]<br>Note: The main concern of this project is developing mathematical modeling of underground storage by using existing models and approaches, risk assessment techniques, and experimental investigations related to chemical and microbial aspects of thermal storage[2]. Moreover, this project also included the feasibility study of storing natural gas, synthetic methane, hydrogen and compressed air in caverns and porous reservoirs and considered economic, political and legal aspects, as well[1].
Underground Sun Storage	Duration: started in 2012[2,1]<br>Contributed country: Austria[2]<br>Participant(s): coordinated by RAG (Rohol-Aufsuchungs Aktiengesellschaft). Other members of the consortium are the University of Leoben (the Department for Agro biotechnology), IFA-Tulln of the University of Natural Resources and Applied Life Sciences, Vienna; the Energy Institute at the Johannes Kepler University Linz; Verbund; and Axiom Angewandte Prozesstechnik GmbH[2]<br>Note: This a feasibility study of storing natural gas or synthesized methane with hydrogen in underground porous reservoirs with regarding to the engineering, economic, and material characteristic issues of UHS. It has been conducted for implementing field test of one storage cycle of the gas mixture containing 10% hydrogen[1]
HyINTEGER	Duration: started in January 2016 till 2019[1]<br>Contributed country: Germany[2,1]<br>Participant(s): the German Federal Ministry of Education and Research (R&D program of Energy Storing), Coordinator: FSU Jena. Partnerships: TU Clausthal, EFZN, GFZ Helmholtz-Centre Potsdam, LEMTA-University of Lorraine, Nancy, and the University of Mainz[2,1]<br>Note: This is the follow-up H2STORE project, so by implementing some experimental tests and numerical simulations and developing analytical methods, assessed the chemical-mineralogical, microbiological and petrophysical-geohydraulic-geomechnical processes in reservoir and cap rocks, and the material behaviour under the corrosive conditions[1]
A French research project	Duration: started in 2011[2]<br>Contributed country: Franc[2]<br>Participant(s): the University of Lorraine and the Carnot Institute ICEEL[2]<br>Note: "Self-organization phenomena in bioreactive multi-component transport through porous media: application to Underground Storage of Hydrogen."[2]
Roads2HyCOM	Participant(s): founded by the European Commission[2]<br>Note: this is a program for large scale hydrogen storage in geological formations[2]
HyLigths	Participant(s): founded by the European Commission[2]<br>Note: this is a program for large scale hydrogen storage in geological formations[2]
US research	Contributed country: US<br>Participant(s): the Sandia National Lab on behalf of the Department of State for Energy in the United States[2]<br>Note: feasibility studies of UHS in geological formations[2]

Table 2. Solubility and Diffusivity values of hydrogen in brine.

No.	Solubility	Temperature	Pressure	Brine Composition	Reference(s)
1	[0-4] mol/m^3	[0-700] ^{o}F	[0-700] psia	pure water	[3]
2	80 mol/m^3	25 ^{o}C	100 bar	pure water	[4]
3	0.784 mol/m^3	25 ^{o}C	1 atm	pure water	[4]
4	37 mol/m^3	30 ^{o}C	50 bar	pure water	[4]
5	[0.001-0.002] g/kg	[0-100] ^{o}F	1 bar	pure water	[4,5]
6	[6-10] $\times 10^{-4}$ mol/kg	[0-30] ^{o}C	1 bar	pure water & 4% NaCl	[4,6]
7	7.9 $\times 10^{-4}$ mol/kg	25 ^{o}C	1 atm	pure water	[7]

No.	Diffusivity (m^2/s)	Hydrogen into	Reference(s)
1	5 $\times 10^{-9}$<br>3 $\times 10^{-11}$	pure water<br>clay	[2,7]
2	10^{-9}<br>10^{-11}	pure water<br>water soaked argillaceous	[8]
3	4.5 $\times 10^{-9}$<br>3 $\times 10^{-11}$	pure water<br>clay	[4]
4	1.6 $\times 10^{-9}$	liquid	[3]

Table 3. Receding and advancing contact angles by digitizing Morrow's graph. * indicates modified values for advancing contact angles which were used in this paper.

θ_i	θ_r	θ_a	modified θ_a^*
22	0	0	0
32	1	20	20
42	2	40	40
51	5	58	58
62	10	81	81
70	15	96	85
75	20	107	85
79	25	115	85
83	30	122	85
86	35	128	85
89	40	134	85
94	50	144	
99	60	153	
103	70	159	
109	80	163	
113	90	167	
118	100	171	
124	110	173	
128	120	175	
133	130	177	
138	140	178	
143	150	179	
148	160	179	
153	170	180	
159	180	180	

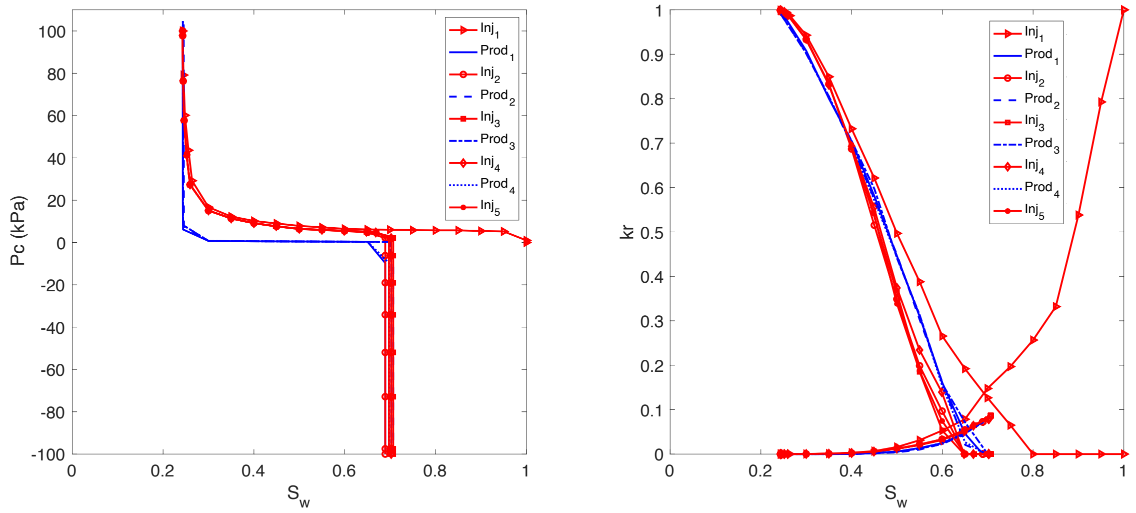

Figure 1. The impact of cyclic transport dynamics on capillary pressure and relative permeabilities for 9 cycles of injection and production of hydrogen into the network of Berea sandstone with advancing and receding contact angles of $\theta_a = 81^o$ and $\theta_r = 10^o$, respectively, using the fluid properties of the base-case.

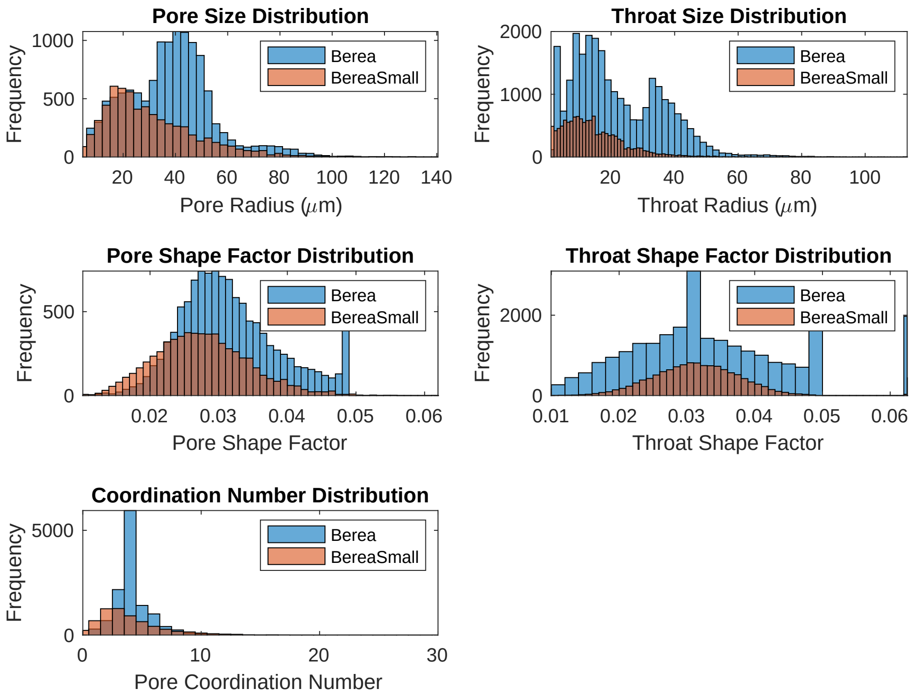

Figure 2. Pore network characteristics for Berea and small Berea.

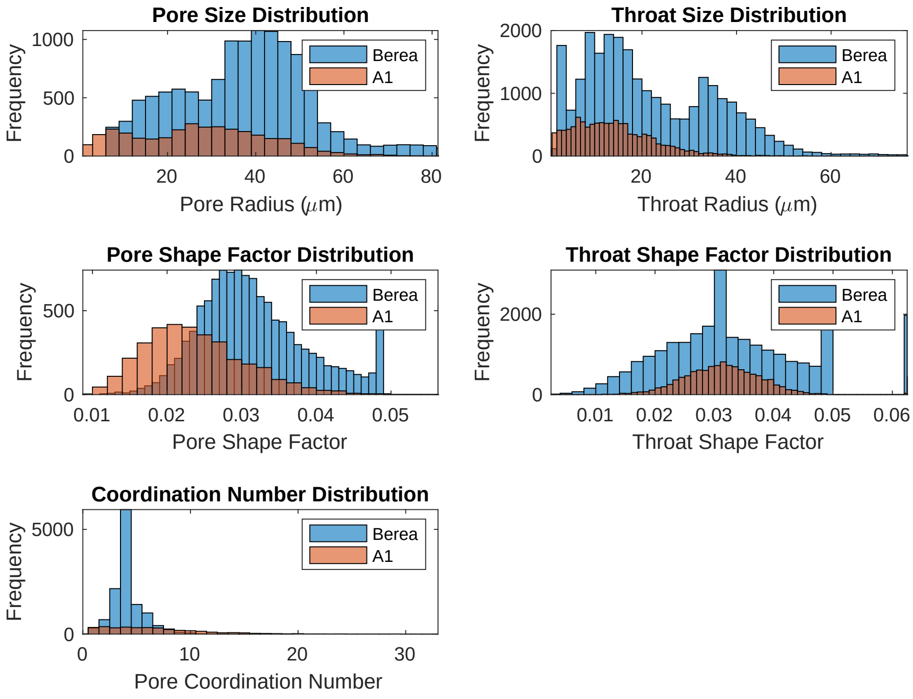

Figure 3. Pore network characteristics for Berea and A1.

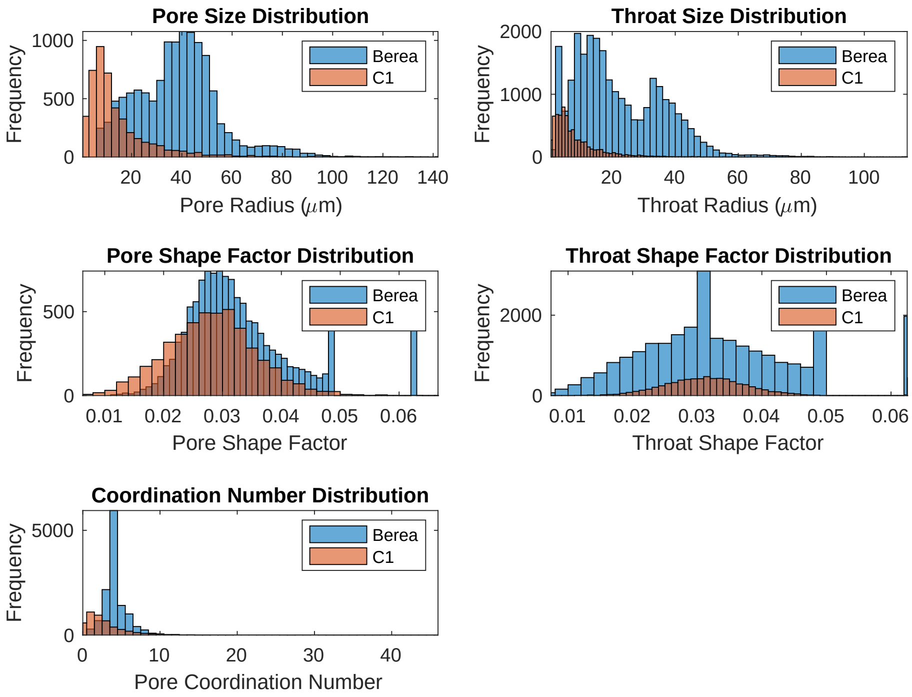

Figure 4. Pore network characteristics for Berea and C1.

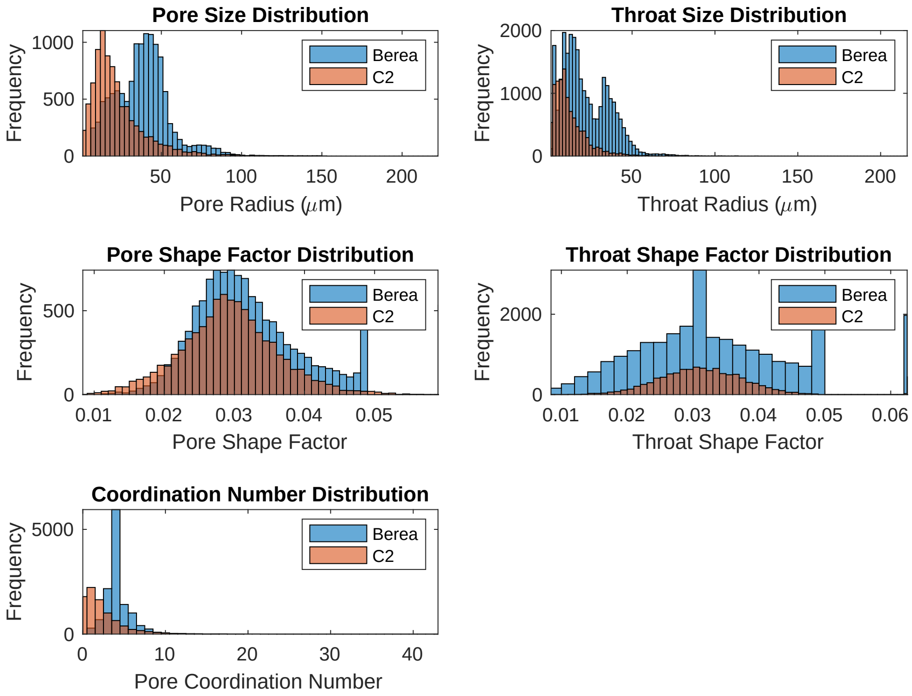

Figure 5. Pore network characteristics for Berea and C2.

Table 4. Used fluid and rock properties for clay volume (H_2-Brine) sensitivity analysis. * indicates the network that was used as the base-case of simulations.

	Test No.	Clay Percentage	Dimensions (mm^3)	No. of pores	No. of throats	Porosity (%)	Permeability (mD)
Rock	1*	0	$3.47 \times 1.39 \times 0.69$	10000	28700	20.78	134.96
	2	10	$3.47 \times 1.39 \times 0.69$	10000	28700	20.78	134.96
	3	20	$3.47 \times 1.39 \times 0.69$	10000	28700	20.78	134.96
	4	30	$3.47 \times 1.39 \times 0.69$	10000	28700	20.78	134.96
	5	40	$3.47 \times 1.39 \times 0.69$	10000	28700	20.78	134.96
	6	50	$3.47 \times 1.39 \times 0.69$	10000	28700	20.78	134.96
Fluid	Phases	$\sigma_{H_2,brine}$ (mN/m)	θ_r (degrees)	θ_a (degrees)	Viscosity ratio	Density difference (kg/m^3)	
	H_2-brine	51	21.56	85	111.745	994.9	

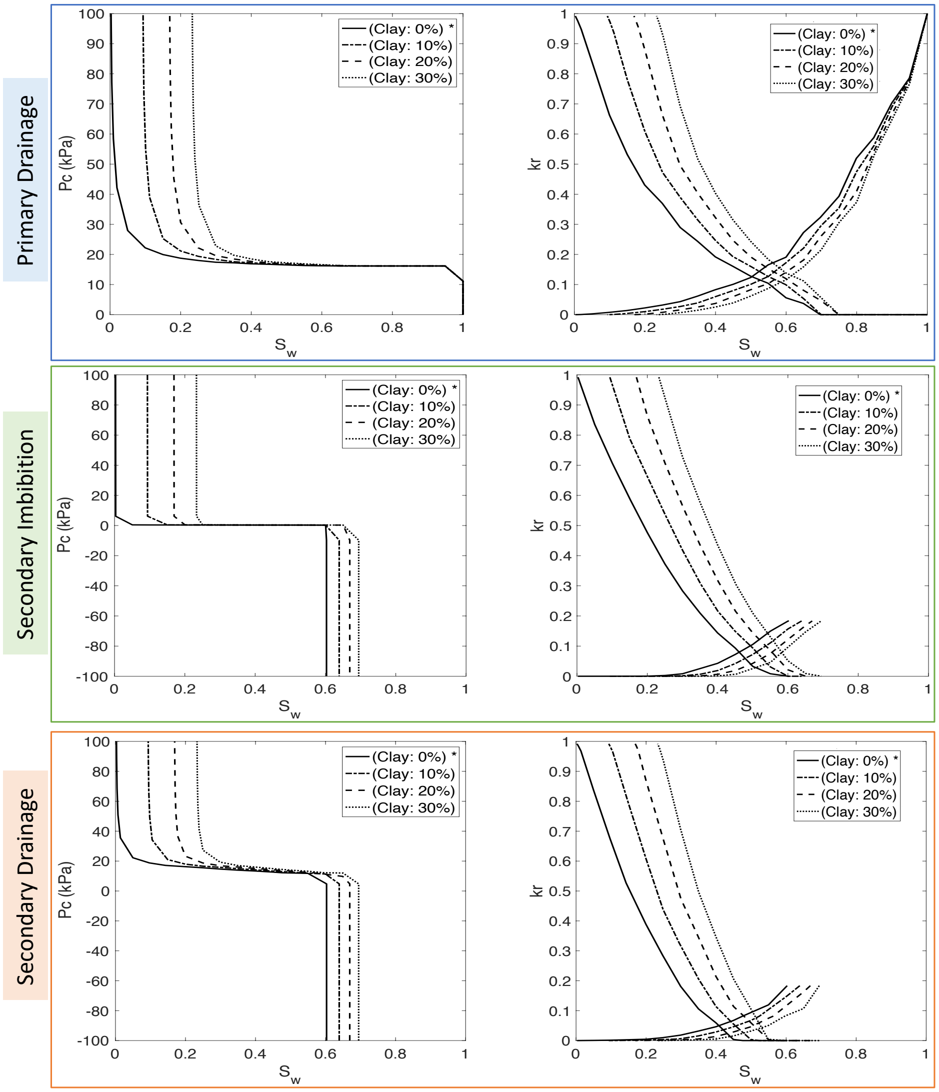

Figure 6. Sensitivity analysis of H_2-brine system on clay volume. * indicates the statistical network that was used as the base-case of simulations.

Table 5. Used fluid and rock properties for coordination number(H_2-Brine) sensitivity analysis. * indicates the network that was used as the base-case of simulations.

	Test No.	Coordination No. (average)	Dimensions (mm^3)	No. of pores	No. of throats	Porosity (%)	Permeability (mD)
Rock	1*	6	$3.46 \times 1.39 \times 0.69$	10000	28700	20.57	135.52
	2	5	$3.47 \times 1.39 \times 0.69$	10000	23936	18.62	77.03
	3	4	$3.45 \times 1.38 \times 0.69$	10000	19302	15.56	30.82
	4	3	$3.47 \times 1.39 \times 0.69$	10000	14794	14.56	8.35
Fluid	Phases	$\sigma_{H_2,brine}$ (mN/m)	θ_r (degrees)	θ_a (degrees)	Viscosity ratio	Density difference (kg/m^3)	
	H_2-brine	51	21.56	85	111.745	994.9	

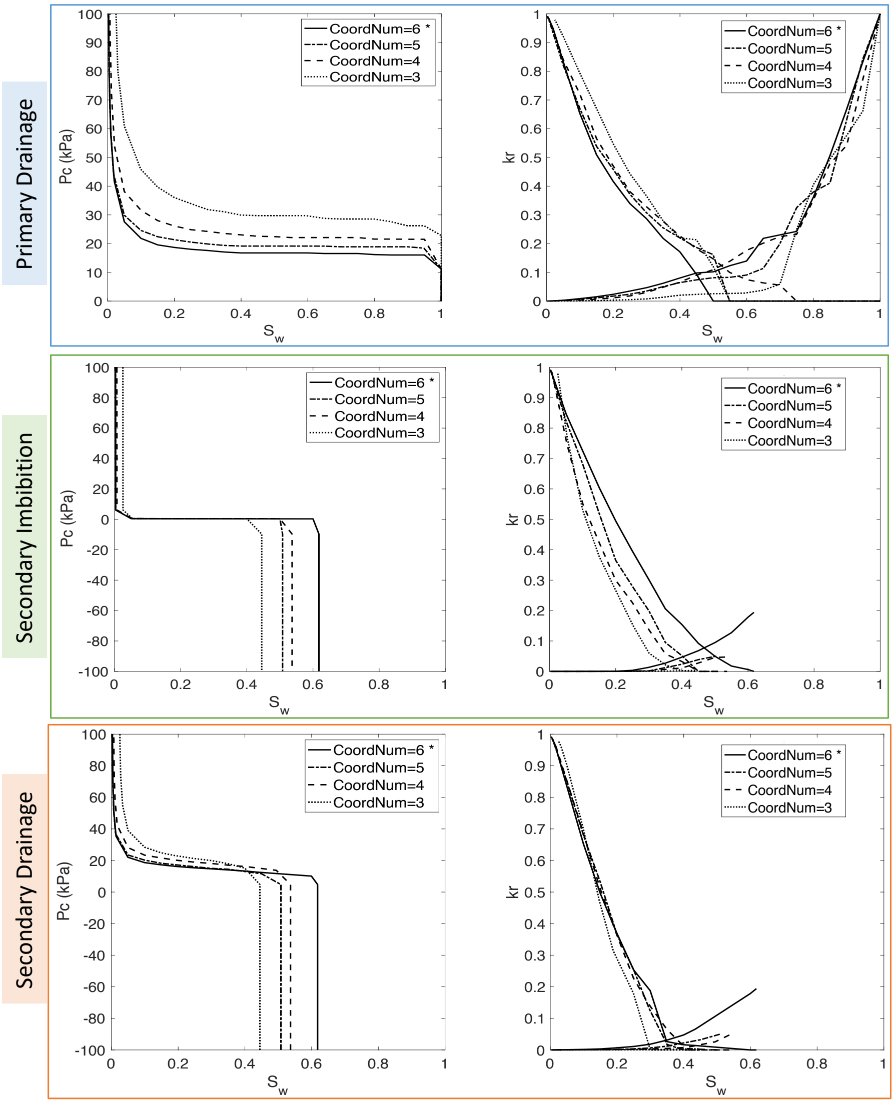

Figure 7. Sensitivity analysis of H_2-brine system on coordination number. * indicates the statistical network that was used as the base-case of simulations.

References

1. Hagemann, B. *Numerical and analytical modeling of gas mixing and bio-reactive transport during underground hydrogen storage*. Phd thesis, Clausthal University of Technology, Germany and the University of Lorraine, France (2017).

2. Panfilov, M. 4 - underground and pipeline hydrogen storage. In Gupta, R. B., Basile, A. & Veziroğlu, T. N. (eds.) *Compendium of Hydrogen Energy*, Woodhead Publishing Series in Energy, 91 – 115, DOI: https://doi.org/10.1016/B978-1-78242-362-1.00004-3 (Woodhead Publishing, 2016).

3. Pichler, M. Assesment of hydrogen - rock interactions during geological storage of ch_4 - h_2 mixtures (2013).

4. Ebrahimiyekta, A. *Characterization of geochemical interactions and migration of hydrogen in sandstone sedimentary formations : application to geological storage*. Phd thesis, Orleans University, France (2017).

5. Lassin, A., Dymitrowska, M. & Azaroual, M. Hydrogen solubility in pore water of partially saturated argillites: Application to callovo-oxfordian clayrock in the context of a nuclear waste geological disposal. *Phys. Chem. Earth, Parts A/B/C* **36**, 1721 – 1728, DOI: https://doi.org/10.1016/j.pce.2011.07.092 (2011). Clays in Natural and Engineered Barriers for Radioactive Waste Confinement.

6. Lucia], M. D., Pilz, P., Liebscher, A. & Kühn, M. Measurements of h2 solubility in saline solutions under reservoir conditions: Preliminary results from project h2store. *Energy Procedia* **76**, 487 – 494, DOI: https://doi.org/10.1016/j.egypro.2015.07.892 (2015). European Geosciences Union General Assembly 2015 - Division Energy, Resources and Environment, EGU 2015.

7. Hemme, C. *Storage of Gases in Deep Geological Structures: Spatial and Temporal Hydrogeochemical Processes Evaluated and Predicted by the Development and Application of Numerical Modeling*. Phd thesis, Clausthal University of Technology, Germany (2019).

8. Tarkowski, R. Underground hydrogen storage: Characteristics and prospects. *Renew. Sustain. Energy Rev.* **105**, 86 – 94, DOI: https://doi.org/10.1016/j.rser.2019.01.051 (2019).
